# Seizure Clusters, Seizure Severity Markers, and SUDEP Risk

**DOI:** 10.3389/fneur.2021.643916

**Published:** 2021-02-12

**Authors:** Manuela Ochoa-Urrea, Nuria Lacuey, Laura Vilella, Liang Zhu, Shirin Jamal-Omidi, M. R. Sandhya Rani, Johnson P. Hampson, Mojtaba Dayyani, Jaison Hampson, Norma J. Hupp, Shiqiang Tao, Rup K. Sainju, Daniel Friedman, Maromi Nei, Catherine Scott, Luke Allen, Brian K. Gehlbach, Victoria Reick-Mitrisin, Stephan Schuele, Jennifer Ogren, Ronald M. Harper, Beate Diehl, Lisa M. Bateman, Orrin Devinsky, George B. Richerson, Guo-Qiang Zhang, Samden D. Lhatoo

**Affiliations:** ^1^National Institute of Neurological Disorders and Stroke Center for Sudden Unexpected Death in Epilepsy Research (CSR), McGovern Medical School, University of Texas Health Science Center at Houston, Houston, TX, United States; ^2^Department of Neurology, McGovern Medical School, University of Texas Health Science Center at Houston, Houston, TX, United States; ^3^Biostatistics & Epidemiology Research Design Core, Division of Clinical and Translational Sciences, McGovern Medical School, University of Texas Health Science Center at Houston, Houston, TX, United States; ^4^University of Iowa Carver College of Medicine, Iowa City, IA, United States; ^5^New York University Langone School of Medicine, New York, NY, United States; ^6^Sidney Kimmel Medical College, Thomas Jefferson University, Philadelphia, PA, United States; ^7^Department of Clinical and Experimental Epilepsy, Institute of Neurology, University College London, London, United Kingdom; ^8^Case Western Reserve University, Cleveland, OH, United States; ^9^Feinberg School of Medicine, Northwestern University, Chicago, IL, United States; ^10^Department of Neurobiology and the Brain Research Institute, University of California, Los Angeles (UCLA), Los Angeles, CA, United States; ^11^Department of Neurology, Cedars-Sinai Medical Center, Los Angeles, CA, United States

**Keywords:** SUDEP, seizure cluster, generalized convulsive seizure, epilepsy, video-EEG (VEEG) monitoring

## Abstract

**Rationale:** Seizure clusters may be related to Sudden Unexpected Death in Epilepsy (SUDEP). Two or more generalized convulsive seizures (GCS) were captured during video electroencephalography in 7/11 (64%) patients with monitored SUDEP in the MORTEMUS study. It follows that seizure clusters may be associated with epilepsy severity and possibly with SUDEP risk. We aimed to determine if electroclinical seizure features worsen from seizure to seizure within a cluster and possible associations between GCS clusters, markers of seizure severity, and SUDEP risk.

**Methods:** Patients were consecutive, prospectively consented participants with drug-resistant epilepsy from a multi-center study. Seizure clusters were defined as two or more GCS in a 24-h period during the recording of prolonged video-electroencephalography in the Epilepsy monitoring unit (EMU). We measured heart rate variability (HRV), pulse oximetry, plethysmography, postictal generalized electroencephalographic suppression (PGES), and electroencephalography (EEG) recovery duration. A linear mixed effects model was used to study the difference between the first and subsequent seizures, with a level of significance set at *p* < 0.05.

**Results:** We identified 112 GCS clusters in 105 patients with 285 seizures. GCS lasted on average 48.7 ± 19 s (mean 49, range 2–137). PGES emerged in 184 (64.6%) seizures and postconvulsive central apnea (PCCA) was present in 38 (13.3%) seizures. Changes in seizure features from seizure to seizure such as seizure and convulsive phase durations appeared random. In grouped analysis, some seizure features underwent significant deterioration, whereas others improved. Clonic phase and postconvulsive central apnea (PCCA) were significantly shorter in the fourth seizure compared to the first. By contrast, duration of decerebrate posturing and ictal central apnea were longer. Four SUDEP cases in the cluster cohort were reported on follow-up.

**Conclusion:** Seizure clusters show variable changes from seizure to seizure. Although clusters may reflect epilepsy severity, they alone may be unrelated to SUDEP risk. We suggest a stochastic nature to SUDEP occurrence, where seizure clusters may be more likely to contribute to SUDEP if an underlying progressive tendency toward SUDEP has matured toward a critical SUDEP threshold.

## Introduction

Seizure clusters occur frequently in patients with epilepsy (PWE). Their frequency ranges from 22–43% in an outpatient setting (1, 2) and occurs in up to 83% in inpatient settings (3). Frequency also depends on the definition used and the population targeted (4). Seizure clusters are usually defined as repetitive and closely grouped seizures whose pattern of occurrence deviates from an expected distribution (5, 6). However, a unified operational definition has not been established (4, 6) Definitions vary from study to study, including: ≥3 seizures in 24 h (1, 2, 4, 7–13), ≥2 in 24 h (14), and 2–4 seizures in <48 h (15). Seizure clusters are associated with an increased risk of status epilepticus and hospitalizations, and are deleterious for quality of life of PWE (7, 16). In addition, seizure clusters are associated with increased mortality and are seen as an adverse event in the epilepsy monitoring unit (EMU) (2, 17, 18).

In the landmark mortality in epilepsy monitoring unit study (MORTEMUS), two or more generalized convulsive seizures (GCS) were captured during video electroencephalography (VEEG) in 7/11 (64%) patients with VEEG monitored SUDEP, and in 3/9 (33%) patients in near-SUDEP (19). Thus, the occurrence of a cluster of GCS has been speculated to be a risk factor for SUDEP. We hypothesized that if GCS clusters are a SUDEP risk factor, electroclinical scrutiny of consecutive GCS may indicate increasing seizure severity. We aimed to determine if there is worsening of electroclinical seizure features from seizure to seizure within a cluster and possible associations between GCS clusters, markers of seizure severity, and SUDEP risk.

## Methods

### Patient Selection

Patients were consecutive, prospectively consented participants in the National Institute of Neurological Disorders and Stroke (NINDS) Center for SUDEP Research's Autonomic and Imaging Biomarkers of SUDEP multicenter project (U01- NS090407), its preliminary phase, the Prevention and Risk Identification of SUDEP Mortality (PRISM) project (P20NS076965) and, prospectively consented participants from Memorial Hermann Hospital Epilepsy Monitoring Unit (Houston, Texas). Patients with drug-resistant epilepsy (failure of adequate trials of ≥2 antiepileptic medications) (20) undergoing VEEG evaluation in the EMU of participating centers from September 2011 until April 2020 were studied. We included patients with VEEG recorded GCS clusters, including generalized tonic clonic seizures, focal to bilateral tonic-clonic seizures, and focal-onset motor bilateral clonic seizures (21). Exclusion criteria were status epilepticus and obscured or unavailable video.

### Data Collection and Definitions

Demographic and clinical data were collected. A GCS cluster was defined as two or more GCS in a 24 h period (14). Epileptogenic zone was classified as generalized (genetic generalized epilepsy in all cases), focal, both, or unknown (22). State of consciousness was defined as either awake or asleep according to Tatum, 2014 (23). Clinical seizure duration was determined by the time between first and last semiological ictal sign, and EEG duration was determined by the time between EEG onset and offset. GCS duration was defined as time from onset of bilateral motor signs of tonicity or clonicity to clinical seizure end. The duration of GCS was further divided into phases according to ictal semiology as previously described (24): a tonic phase, a jittery phase (also called vibratory period) and a clonic phase. In addition, tonic phase semiology was classified into 4 categories, based on a modified classification proposed by previous authors, (25) in ictal decerebrate, decorticate, hemi-decerebrate and absence of tonic phase. Early nursing intervention was defined as oxygen administration or suction applied during the seizure or within 5 s of seizure termination (25). The impact of anti-seizure medication (ASM) changes on electroclinical features was assessed. ASM changes were collected as tapering (gradual decrease in medication), withdrawal (complete cessation of medication), increase or resumption of medication, and no change. Administration of rescue medication was determined as the administration of IV benzodiazepines and/or IV bolus of ASM during or after a seizure.

Patients underwent prolonged surface VEEG monitoring with the 10–20 international electrode system or invasive VEEG with subdural electrodes, depth electrodes or stereoEEG. Peripheral capillary oxygen saturation (SpO_2_) and heart rate were monitored. SpO_2_ <90% was considered hypoxemia. Breathing rate was assessed between 2 min pre-ictally and 3 min after clinical seizure end through careful composite analysis of inductance plethysmography, EEG breathing artifact, visually inspected thoracoabdominal excursions, and auditory breathing information. Central apnea was defined as ≥1 missed breaths without any other explanation (i.e., speech, movement, or intervention). Ictal central apnea (ICA) was defined as apnea during non-convulsive seizure or apnea occurring in the pre-convulsive phase of GCS (26). Post-convulsive central apnea (PCCA) referred to apnea after a GCS (26). Postictal generalized EEG suppression (PGES) was defined according to Lhatoo et al. (27). Presence and duration of postictal generalized EEG suppression (PGES) were determined by visual analysis. Presence and duration of postictal EEG burst suppression were also determined. Combined PGES and burst suppression, following PGES, made up the EEG recovery duration. Heart rate variability (HRV) was assessed using the LabChart HRV module (LabChart Pro 8 software; ADInstruments, Sydney, Australia). For a detailed description of the data collection and definitions please refer to the Supplementary Material.

### Statistical Analysis

Summary statistics were reported as mean ± standard deviation (SD), median with range, and frequency with percentage. For continuous outcomes, linear mixed effects models were used to study the difference between the first and subsequent seizures while accounting for the within-cluster correlation. Empirical covariance estimators were used for robust inferences. For binary outcomes, generalized linear mixed effects models were used, with binary distribution and logit link. Each model was adjusted for sex, age, epilepsy duration, epileptogenic zone, early nursing intervention (early O_2_ administration and suction) and administration of rescue medication. Because the percentage of clusters composed of five and seven seizures was low, we performed the mixed model until the 4th seizure and excluded the 5th, 6th, and 7th seizures from the analysis. The level of significance was set at *p* < 0.05. Statistical analysis was done with SAS software version 9.4 (Cary, NC).

## Results

One hundred and twelve GCS clusters were identified in 105 patients with 285 seizures. Three patients had two clusters and two patients had three clusters during different EMU admissions. The remainder had one cluster only. Thirteen (11.6%) clusters occurred during invasive EEG. Mean age was 35.9 ± 14 years (median 33, range 12–69), 47% were female, and mean epilepsy duration was 16.3 ± 11.6 (14, 0.08–43) years. Fifty-six (53.33%) patients had ≥3 GCS per year, and 35 (33.33%) had <3 GCS per year. Sixteen (15.2%) patients had a history of seizure clusters. Fifty-nine percent of seizures occurred during sleep. The mean inter-seizure interval was 6.52 h ± 6.12 (3.78, 0.05–23.62). The epileptogenic zone was most frequently temporal lobe, followed by frontal lobe (Table 1). Average clinical seizure duration was 93.6 ± 44.7 s (84.5, 28–393), average EEG seizure duration was 105.7 s ± 50.5 s (94, 38–516) and the GCS phase lasted on average 48.7 ± 19 s (49, 2–137). PGES was present in 184 (64.6%) seizures, lasting on average 36.84 s ± 20.98 (35, 2–135) and PCCA was present in 38 (13.33%) seizures lasting 10.74 s ± 6.99 (8, 3–26). Electroclinical seizure features and their respective durations are shown in Table 2.

**Table 1:** Epileptogenic zone.

**Variable**	**Patients, *n* = 105 (%)**	**Cluster, *n* = 112 (%)**
Epileptogenic zone		
Generalized	10 (9.5)	10 (8.9)
Focal	93 (88.6)	100 (89.3)
Temporal	37 (39.8)	38 (38)
Bitemporal	13 (13.9)	13 (13)
Frontal	19 (20.4)	22 (22)
Parietal	1 (1)	1 (1)
Insula	1 (1)	1 (1)
Multifocal	13 (13.9)	13 (13)
Lateralized	9 (9.7)	12 (12)
Unknown	2 (1.9)	2 (1.8)
Lateralization of epilepsy		
Left	40 (38.1)	43 (38.4)
Right	26 (24.8)	29 (25.9)
Generalized	10 (9.5)	10 (8.9)
Bilateral	27 (25.7)	28 (25)
Unknown	2 (1.9)	2 (1.8)

*n, number*.

**Table 2:** Seizure features and their durations.

**Seizure feature**	**Seizures *n* = 285 (%)**	**Average duration (s)**	**SD (s)**	**Range (s)**
Tonic phase	181 (63.5)	7.66	3.96	(2, 22)
Jittery phase	230 (80.7)	10.30	7.50	(1, 41)
Clonic phase	284 (99.6)	37.08	17.09	(5, 130)
Decerebration	130 (45.6)	16.07	11.58	(1, 54)
Decortication	65 (22.8)	15.89	11.19	(2, 50)
PGES	183 (64.2)	36.84	21.04	(2, 135)
Hypoxemia postGCS	84 (29.5)	83.31	46.71	(4, 310)
EEG recovery	185 (64.9)	74.88	60.46	(2, 354)
PCCA	35 (12.3)	10.74	7.09	(3, 37)
ICA	72 (38.9)	17.54	12.95	(5, 74)

*s, seconds; n, number; PGES, post-ictal generalized electroencephalographic suppression; EEG, electroencephalographic; PCCA, post-convulsive central apnea; ICA, ictal central apnea*.

There were 70 clusters (66.67%) with two seizures, 30 (28.57%) with three seizures, six clusters (5.71%) with four seizures, five clusters (4.76%) with five seizures and one cluster (0.95%) with seven seizures.

### Electroclinical Characteristics

**Figure 1:**
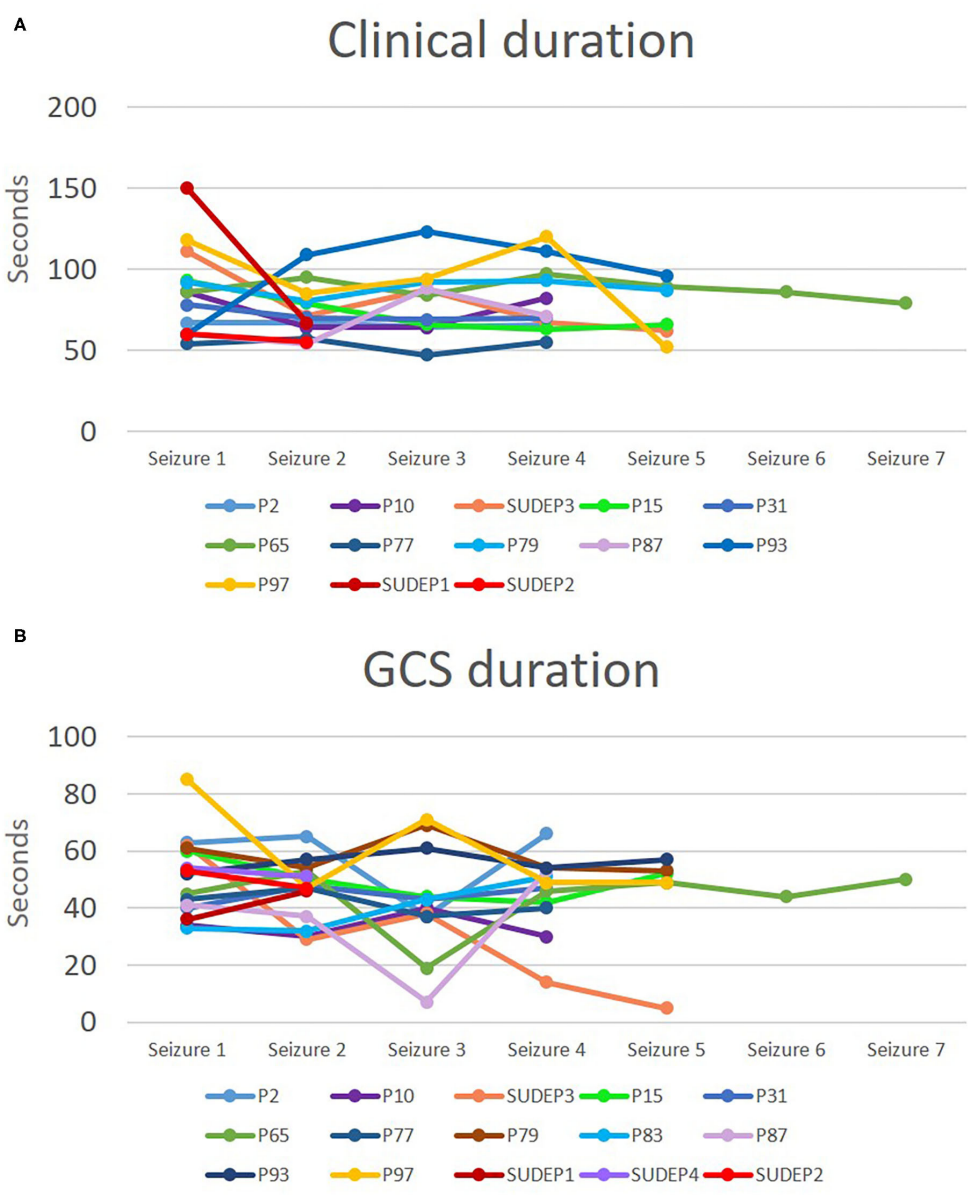
Clinical seizure and convulsive phase durations in patients with at least four seizures in a cluster and for SUDEP cases. Durations for clinical seizure **(A)** and for GCS **(B)** are depicted. Each line represents a cluster and each point represents the duration in seconds for a specific seizure in the cluster.

We did a group analysis to compare electroclinical features of the first seizure with subsequent seizures, adjusting for sex, age, epilepsy duration, epileptogenic zone, administration of rescue medication, early administration of oxygen and early suctioning (early nursing intervention). No significant differences were found in clinical or EEG seizure duration, tonic phase duration, GCS duration, PGES duration and EEG recovery (Table 3). Concerning HRV, there were no statistically significant differences between seizures. In addition, state of consciousness (awake or asleep), SpO_2_ recovery, presence of decerebrate posturing, total hypoxemia duration, and presence of PGES did not show differences between seizures. Seizure-to-seizure comparisons of clinical seizure durations and GCS durations showed apparently random variations representing either improvement, deterioration or no change. We depicted the variation in seizure duration for clinical seizure duration and GCS duration as an example (Figure 1). As inferred from the graphs in the figure, seizures tended to have the same or random durations throughout the cluster.

**Table 3:** Comparison of electroclinical features of first seizure with each subsequent seizure in a cluster.

**Seizure feature**	**Seizure #**	**Estimate**	**Standard Error**	***p-*value**
Clinical duration	1	0		
	2	1.91	5.39	0.725
	3	−0.62	5.51	0.911
	4	2.04	6.96	0.770
EEG duration	1	0		
	2	−1.57	7.05	0.824
	3	0.48	7.24	0.948
	4	−0.49	7.97	0.951
Decerebration duration	1	0		
	2	1.06	1.21	0.386
	3	0.79	2.10	0.710
	4	7.22	1.15	**<0.0001**
Tonic phase duration	1	0		
	2	0.75	0.60	0.219
	3	1.87	1.07	0.087
	4	5.96	3.15	0.064
Clonic phase duration	1	0		
	2	−2.19	2.54	0.390
	3	−6.05	3.17	0.059
	4	−11.87	3.63	**0.002**
GCS duration	1	0		
	2	−0.62	2.35	0.792
	3	−2.68	2.97	0.369
	4	−5.41	3.86	0.164
PGES duration	1	0		
	2	−2.35	3.77	0.535
	3	−5.43	3.69	0.147
	4	−10.98	17.26	0.527
EEG recovery	1	0		
	2	−18.63	10.81	0.091
	3	−11.54	16.29	0.482
	4	−49.71	26.09	0.062
PCCA duration	1	0		
	2	−2.13	4.30	0.654
	3	0.94	1.63	0.606
	4	−7.49	1.70	**0.022**
ICA duration	1	0		
	2	3.16	2.72	0.255
	3	5.42	4.14	0.201
	4	10.79	3.53	**0.005**

*EEG, electroencephalographic; GCS, generalized convulsive seizure; PGES, post-ictal generalized electroencephalographic suppression; PCCA, post-convulsive central apnea; ICA, ictal central apnea. Bold values indicate p <0.05*.

Duration of clonic phase and PCCA duration were significantly shorter in the 4th seizure as compared to the first one (Table 3), *p* = 0.002 and *p* = 0.022, respectively. On the other hand, duration of decerebrate posturing (*p* < 0.0001) and ictal central apnea (ICA) (*p* = 0.005) were longer in the 4th seizure. With both parameters we found a progressive increase in duration with subsequent seizures.

### Anti-seizure Medications

For 217 (76%) seizures we had information on changes in anti-seizure medications (ASM) during the EMU admission. Of these, 80 (36.9%) seizures occurred during medication taper, 68 (31.3%) during medication withdrawal, 66 (30.4%) during resumption or increase of ASM and three (1.38%) seizures occurred with no change in ASM. We grouped the changes in ASM into those who had taper or withdrawal and those who had increase or no change. We compared both groups and found no statistically significant differences with regards to electroclinical features (Supplementary Table 1). However, the group with taper or withdrawal of ASM were less likely to have PGES. Rescue medication, either a benzodiazepine, an intravenous bolus of ASM or both, was given after 34 seizures in 25 clusters.

### SUDEP Cases

Four cases of definite/probable SUDEP were established during follow-up. They died between 9 months and 5.5 years after admission. Two of them were found unresponsive in bedroom and for the other two there is no current information about the circumstances of death. Of these cases, only one had a known history of seizure clusters before the EMU evaluation. Average age was 49.25 ± 10.15 years (51.5, 33–61) with a duration of epilepsy of 34.5 ± 7.5 years (35.5, 24–43). They had between 12 and 24 GCS in the prior year. There were no consistent electroclinical characteristics among them. However, two had PGES >50 s. The mean intra-cluster inter-seizure interval for SUDEP patients was 5.14 ± 5.02 h (3.24, 1.63–14.82). In comparison with patients who did not die, there was no difference in the inter-seizure interval (*p* = 0.59).

## Discussion

**Figure 2:**
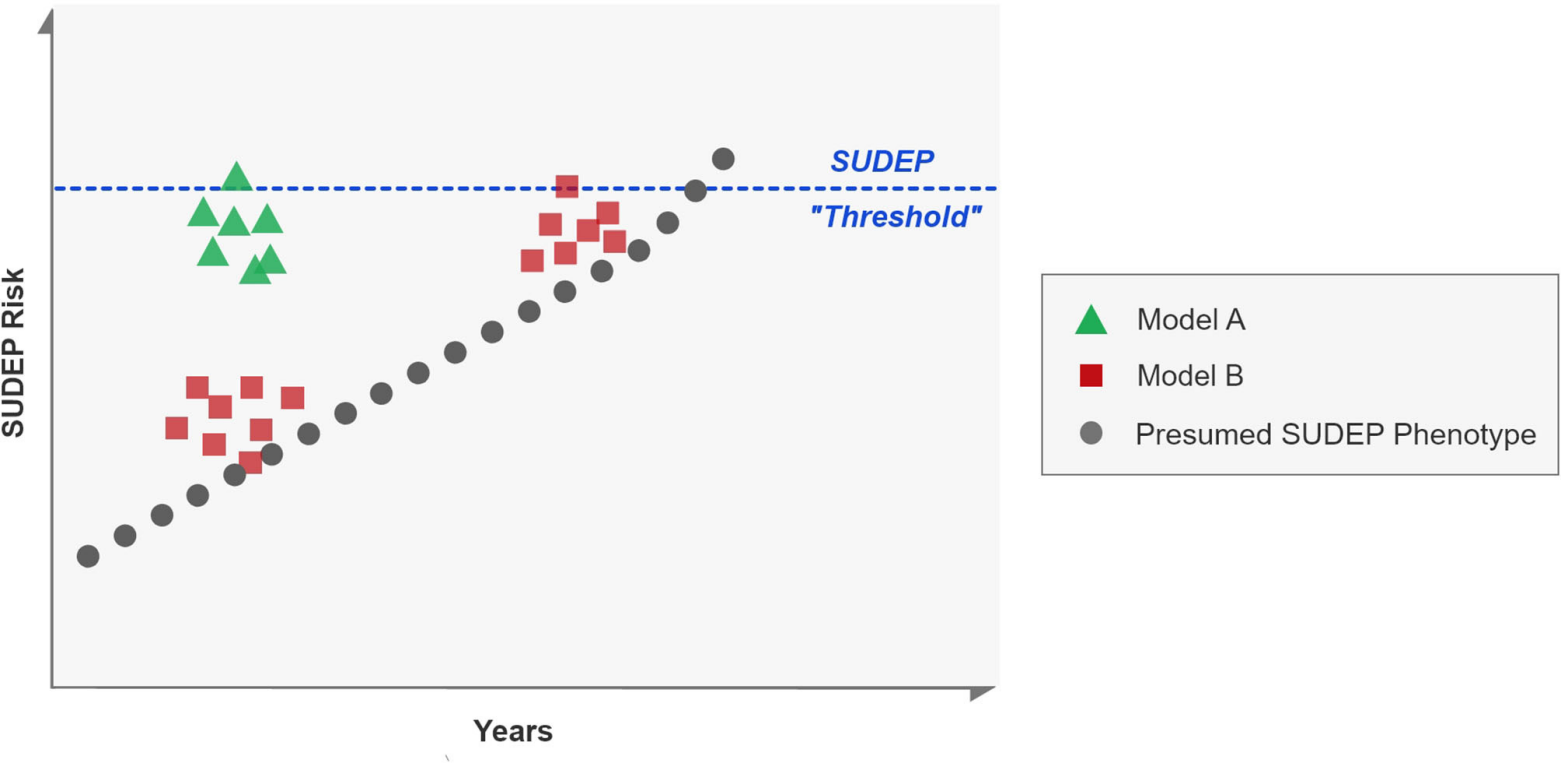
Models of seizure clusters and SUDEP risk. Each square, dot or triangle represents a seizure. A proposed SUDEP phenotype is represented in black, where sporadic seizures occur over years, eventually culminating in SUDEP. Model A proposes that clusters are important for risk of SUDEP, regardless of underlying tendency whereas Model B denotes that risk is only enhanced if the underlying risk has also progressed toward a hypothetical SUDEP “threshold.” Our data suggests that model B may be more likely than model A.

Our prospective study of GCS in the EMU suggest that the markers of seizure severity in seizure clusters, in each consecutive seizure, change randomly. Some markers show improvement while others either stay the same or deteriorate with subsequent seizures. This heterogeneity suggests that clusters alone may not predispose to SUDEP, but that their timing in relation to the overall course of a patient's epilepsy may be more important for mortality risk. Thus, the course of SUDEP may be a stochastic, rather than probabilistic process, where an underlying progressive increase in SUDEP risk accrues over time. Seizure clusters early in this progression pose less risk than clusters late in the process (Figure 2, model B). The MORTEMUS study suggested that seizure clusters were important premortem phenomena (19), although this was not a controlled observation, and no electroclinical characterization of seizure clusters was carried out. Our study suggests that seizure clusters in themselves may not be risk factors for death. Known risk factors of younger age of onset of epilepsy and longer duration of epilepsy may indicate that the underlying tendency toward SUDEP is a progressive phenomenon (28–30). We found no significant effect of duration of epilepsy and age of onset of epilepsy on tendency to worsening of electroclinical markers of seizure severity. Supporting the notion that clusters may not be related to SUDEP risk is the PCDH19-Related Epilepsy Syndrome. It is a childhood onset epilepsy syndrome characterized by seizure clusters (31). To the best of our knowledge, no case of SUDEP related to PCDH19 has been reported in the literature.

The four prospectively ascertained SUDEP patients in our study had very long durations of epilepsy (24–43 years), and the time from recorded clusters to SUDEP ranged from 9 months to 5.5 years. None of the patients were reported to have seizure clusters around the time of SUDEP. Two patients had a very high frequency of GCS (up to 24 per year) and two had prolonged PGES (>50 s); both have been proposed as risk factors for SUDEP (27, 32). However, the clusters in our SUDEP cases behaved very similarly to clusters in the rest of our study population.

GCS are the most important risk factor for SUDEP (32). A recent study suggested that GCS become dramatically lethal when they occur under circumstances such as in night time, sleeping alone, living alone and prone position (33). In addition, if risk factors such as living alone and the presence of GCS could be modified, a great proportion (69%) of SUDEP cases could be prevented (34). These observations suggest that GCS clusters occurring in well-supervised environments such as the EMU, like in this study, are unlikely to cause SUDEP.

On examination of electroclinical seizure features, we found no progressive increase in clinical or electrographic seizure duration. There was also no increase in various seizure severity markers such as tonic phase and convulsive phase durations, presence of decerebrate posturing, hypoxemia duration, SpO_2_ recovery, and presence of PGES. Some patients showed no significant changes in seizure features from seizure to seizure, whereas others either improved or deteriorated. Within individuals, each subsequent seizure could either show improvement or deterioration in seizure severity, suggesting random variations.

On the other hand, in grouped analysis, durations of clonic phase and PCCA were significantly shorter in subsequent seizures. These findings may indicate faster recovery with subsequent seizures, from a clinical and respiratory standpoint, and thus an adaptive process that is perhaps protective against SUDEP risk. It is possible that seizure termination mechanisms are enhanced with repetitive seizures (35, 36), and lead to faster recovery of respiratory drive and to a shorter clonic phase. However, in contrast to a previous study that found the last seizure in a cluster to be longer, we failed to find significant differences in terms of clinical and EEG seizure duration between the first and the fourth seizure (3) (Figure 1). The difference in these findings could reflect dissimilarities in seizure cluster definition and the type of seizures explored. We only included GCS in our study, while other studies have included non-convulsive seizures within the cluster (3). We chose to include only GCS since these events are directly related to SUDEP risk (32, 37). In addition, a study found that in patients who later developed SUDEP, heart rate tended to increase with subsequent seizures in a clusters (38). We evaluated HRV and we did not find any changes in these parameters with subsequent seizures within a cluster.

Interestingly, in grouped analysis, we found some seizure features to worsen, namely duration of decerebrate posturing and ICA duration. Both decerebrate posturing duration and ICA duration are putative markers of seizure severity (39, 40). Decerebrate posturing has been associated with PGES, a proposed SUDEP biomarker (40). Similarly, prolonged ICA (>60 s) has been associated with severe hypoxemia (39). Although, ICA was longer with each seizure, the average duration of ICA was short (17.54 s). Thus, a much larger number of seizure clusters and patients may be needed for study to throw light on the apparently contradictory improvements in some seizure features and deterioration in others.

With regards to ASM, we found that tapering or withdrawal did not influence electroclinical features of a seizure. PGES was more likely to happen in the group with increase or no change in medication. However, PGES duration did not show any difference.

Limitations of our study include difficulty in using a standardized definition of seizure clusters since no consensus currently exists. We chose the definition of two or more GCS in 24 h since the MORTEMUS study observed that patients with SUDEP had two or more seizures before death (19). Clusters in our sample were created by medication discontinuation in the EMU and so they may not behave as seizure clusters in ambulatory PWE. Thus, these may be biologically distinct phenomena. In addition, we did not include in the analysis non-convulsive seizures. Hence, it is not possible to ascertain whether other seizure types could potentially affect the severity of physiologic changes seen in a cluster that also include GCS. We acknowledge that there was a low number of SUDEP cases in our sample.

## Conclusions

If there is no evidence of convincing, progressive deterioration of seizure severity parameters with subsequent seizures, then, is there a role for clusters in SUDEP? The findings of this study suggest that the occurrence of clusters alone does not set the stage for SUDEP with each successive seizure, in quantifiable terms. We therefore hypothesize that SUDEP may be the culmination of underlying disease progression (41) that reaches a critical SUDEP threshold; seizure clusters may only become important for enhanced mortality risk if they occur around this threshold.

In conclusion, clustering failed to show evidence of clinical or electroencephalographic deterioration with each seizure that would explain why seizure clusters may lead to SUDEP, as found by the MORTEMUS study (19). Although clusters may reflect epilepsy severity, their occurrence by themselves may be unrelated to SUDEP risk, and may require underlying progression toward a SUDEP threshold before finally leading to SUDEP.

## Data Availability Statement

The raw data supporting the conclusions of this article will be made available by the authors, without undue reservation.

## Ethics Statement

The studies involving human participants were reviewed and approved by Case Western Reserve University and University Hospitals, as part of NIH/NINDS Center for SUDEP Research study (U01 NS090405, U01 NS090406, U01 NS090407). Written informed consent to participate in this study was provided by the participants' legal guardian/next of kin.

## Author Contributions

MO-U: major role in the acquisition and analysis of data, design and conceptualized the study, interpreted the data, and drafted the manuscript for intellectual content. NL and SL: design and conceptualized study, interpreted the data, and revised the manuscript for intellectual content. LV, SJ-O, MR, JPH, MD, JH, NH, VR-M, CS, JO, and ST: data acquisition. LZ: analysis of data, statistical analysis, and reviewed the manuscript for intellectual content. RS, DF, MN, LA, BG, SS, RH, BD, LB, OD, GR, and G-QZ: revised the manuscript for intellectual content.

## Conflict of Interest

The authors declare that the research was conducted in the absence of any commercial or financial relationships that could be construed as a potential conflict of interest.
