# Supplementary material for: Seizure Clusters, Seizure Severity Markers, and SUDEP Risk
Source: Front Neurol. 2021 Feb 12;12:643916. doi: 10.3389/fneur.2021.643916 (PMC7907515; doi:10.3389/fneur.2021.643916)
Supplement: Supplementary file 1 [file Table_1.DOCX]

Supplementary Material

# Methods.

**Data collection and definitions**

Demographic and clinical data were collected, including phenotypic epilepsy characteristics, epilepsy duration, semiological features and awake or asleep states prior to each seizure. A GCS cluster was defined as 2 or more GCS in a 24 hour period (14). Epileptogenic zone, gathered from the analysis of electroencephalographic (EEG), imaging and clinical data, was classified as generalized (genetic generalized epilepsy in all cases), focal, both, or unknown (22). State of consciousness was defined as either awake (determined by the presence of posterior dominant rhythm, muscle artifact, eye blinking artifact on EEG) or asleep (23). Sleep was defined by VEEG according to Tatum, 2014 (23). Clinical seizure duration was determined by the time between first and last semiological ictal sign, and EEG duration was determined by the time between EEG onset and offset. GCS duration was defined as time from onset of bilateral motor signs of tonicity or clonicity to clinical seizure end. The duration of GCS was further divided into phases according to ictal semiology as previously described (24): a tonic phase, a jittery phase (also called vibratory period) and a clonic phase. In addition, tonic phase semiology was classified into 4 categories, based on a modified classification proposed by previous authors (25): 1) Ictal decerebrate posturing: bilateral symmetric tonic arm extension, 2) Ictal decorticate posturing: bilateral symmetric tonic arm flexion without progression to decerebration, 3) Ictal hemi-decerebrate posturing: tonic extension of one arm with flexion of contralateral arm without progression to decorticate or decerebrate posturing, and 4) absence of ictal tonic phase. Early nursing intervention was defined as oxygen administration or suction applied during the seizure or within 5 seconds of seizure termination (25). The impact of anti-seizure medication (ASM) changes on electroclinical features was assessed. ASM changes were collected as tapering (gradual decrease in medication), withdrawal (complete cessation of medication), increase or resumption of medication, and no change. Administration of rescue medication was determined as the administration of IV benzodiazepines and/or IV bolus of ASM during or after a seizure.

Patients underwent prolonged surface VEEG monitoring with the 10–20 international electrode system or invasive VEEG with subdural electrodes, depth electrodes or stereoEEG. EEG and electrocardiography (ECG) were acquired using Nihon Kohden (Tokyo, Japan) or Natus (Pleasanton, California, USA), or Micromed (Modigliani Veneto, Italy) acquisition platforms. Peripheral capillary oxygen saturation (SpO_2_) and heart rate were monitored with pulse oximetry and plethysmography (Nellcor OxiMax N-600x, Covidien, MN). SpO_2_ less than 90% was considered hypoxemia. Chest wall and abdominal excursions were recorded with inductance plethysmography (Ambu, Ballerup, Denmark; and Sleepmate or Perfect Fit 2, Dymedix, St. Paul, MN). Breathing rate was assessed between 2 minutes pre-ictally and 3 minutes after clinical seizure end through careful composite analysis of inductance plethysmography, EEG breathing artifact, visually inspected thoracoabdominal excursions, and auditory breathing information. Central apnea was defined as ≥1 missed breaths without any other explanation (i.e., speech, movement, or intervention). Ictal central apnea (ICA) was defined as apnea during non-convulsive seizure or apnea occurring in the pre-convulsive phase of GCS (26). Post-convulsive central apnea (PCCA) referred to apnea after a GCS (26). Postictal generalized EEG suppression (PGES) was defined as the immediate postictal (within 30 seconds), generalized absence of electroencephalographic activity greater than 10µV in amplitude, allowing for muscle, movement, breathing, and electrode artifacts (27). Presence and duration of postictal generalized EEG suppression (PGES) were determined by visual analysis. Presence and duration of postictal EEG burst suppression were also determined. Combined PGES and burst suppression, following PGES, made up the EEG recovery duration. Heart rate variability (HRV) was assessed using the LabChart HRV module (LabChart Pro 8 software; ADInstruments, Sydney, Australia); the analyses were performed both in the time and frequency domains 5 minutes preictal and 5 minutes postictal in all seizures, except for 42 seizures in which HRV was calculated in 2 to 4 minutes due to low quality signal. The following HRV parameters were assessed: standard deviation of RR intervals (SNRR), root mean square of successive RR interval differences (RMSSD), percentage of successive RR intervals that differed by more than 50 milliseconds (pNN50), low frequency power and high frequency power.

# Table 1

| **Seizure feature** | **Estimate** | **Standard Error** | **p value** |
| --- | --- | --- | --- |
| Clinical duration | 1.47 | 7.20 | 0.839 |
| EEG duration | 3.86 | 8.06 | 0.633 |
| Decerebration duration | 0.54 | 1.41 | 0.705 |
| Tonic phase duration | -0.47 | 1.51 | 0.754 |
| Clonic phase duration | 5.27 | 3.00 | 0.082 |
| GCS duration | 3.64 | 2.59 | 0.163 |
| PGES duration | 4.27 | 3.97 | 0.287 |
| EEG recovery | -1.77 | 10.36 | 0.865 |
| Hypoxemia duration | 18.90 | 9.40 | 0.054 |
| PCCA duration | -1.18 | 0.94 | 0.256 |
| ICA duration | -0.07 | 2.32 | 0.975 |
| SpO2 recovery duration | 4.09 | 6.52 | 0.536 |
| Presence of decerebration | 0.07 | 0.44 | 0.872 |
| Presence of PGES | -0.81 | 0.40 | **0.044** |
| Presence of PCCA | -0.77 | 0.68 | 0.254 |

Linear mixed effects model was used to compare the group anti-seizure medication (ASM) increased or unchanged versus the group tapering or withdrawal of ASM. EEG= electroencephalographic; GCS= generalized convulsive seizure; PGES= post-ictal generalized electroencephalographic suppression; PCCA= post-convulsive central apnea; ICA= ictal central apnea; SpO2 = Peripheral capillary oxygen saturation.
